# Osteochondroma Arising from the Thyroid Cartilage: A Case Report and Literature Review

**DOI:** 10.1155/2021/8286012

**Published:** 2021-09-02

**Authors:** Jessa E. Miller, Shaghauyegh S. Azar, Dinesh K. Chhetri

**Affiliations:** ^1^University of California, Department of Head and Neck Surgery, Los Angeles, CA, USA; ^2^University of California, David Geffen School of Medicine, Los Angeles, CA, USA

## Abstract

**Introduction:**

Osteochondromas are relatively common benign bone tumors often located at the metaphyseal ends of long bones; however, they are rare in the head and neck region. The objective of this study is to present a case of an osteochondroma arising from the thyroid cartilage causing insidious dysphonia and to present a literature review.

**Methods:**

The medical record of a patient treated for osteochondroma of the thyroid cartilage was reviewed. A literature search on osteochondromas was conducted using PubMed and Google Scholar. The epidemiology, presentation, diagnosis, treatment, and outcomes of osteochondromas were reviewed.

**Results:**

A 50-year-old female presented with nine months of dysphonia and aphonic voice breaks. Laryngovideostroboscopy revealed a left false vocal fold fullness, glottal gap, and vibratory phase asymmetry. A CT neck demonstrated a well-circumscribed 5 × 8 × 9 mm mass arising from the left thyroid cartilage lamina with a thin calcified rim and a heterogeneous decreased attenuation center. The tumor was excised surgically. Histopathologic analysis demonstrated hyaline cartilage overlying lamellar bone with fatty bone marrow, consistent with osteochondroma. English language literature review revealed no cases of osteochondroma of the thyroid cartilage. The presenting features of osteochondroma may depend on the size and location of the lesion. It is critical to differentiate between benign and malignant bone tumors, and physicians must rely on their clinical examination, radiographic findings, and histopathologic analysis to make the correct diagnosis.

**Conclusions:**

Osteochondromas of the laryngeal framework are extremely rare, and to our knowledge, there have been no reports in the literature of this tumor arising from the thyroid cartilage. Dysphonia may be the presenting symptom in a patient with a thyroid cartilage mass causing restricted mobility of the true vocal folds.

## 1. Introduction

Osteochondroma, also referred to as osteocartilaginous exostosis, is a benign, cartilage-forming tumor that is typically located at the metaphyseal ends of long bones, most commonly in the femur and humerus [1]. Osteochondromas make up approximately 30% of benign bone tumors and typically present during childhood [1, 2]. Although there is a reported association with radiation therapy, these tumorstypically arise spontaneously [3]. The risk for malignant transformation of osteochondromas is less than 1%; however, in patients with multiple hereditary osteochondromatosis, an autosomal dominant disorder caused by *EXT1* or *EXT2* gene mutations, this risk increases to 1–20% [1]. In this report, we describe a rare case of osteochondroma arising from the thyroid cartilage. To our knowledge, this is the only reported case of osteochondroma of the thyroid cartilage to date.

## 2. Case Presentation

**Figure 1:**
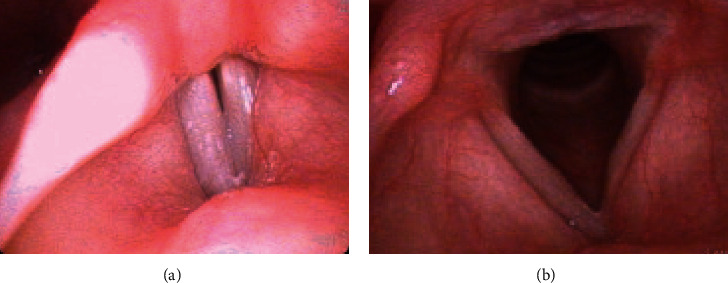
Laryngovideostroboscopy exam showing (a) fullness of the left false vocal fold compared to the right with mild posterior glottal gap and (b) subtle fullness of the left true vocal fold.

**Figure 2:**
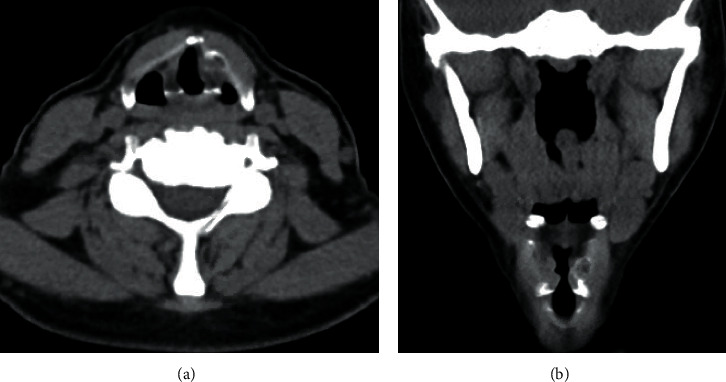
Axial (a) and coronal (b) CT without contrast demonstrating a mass arising from the left thyroid cartilage lamina with a thin calcified rim and a heterogeneous decreased attenuation center.

**Figure 3:**
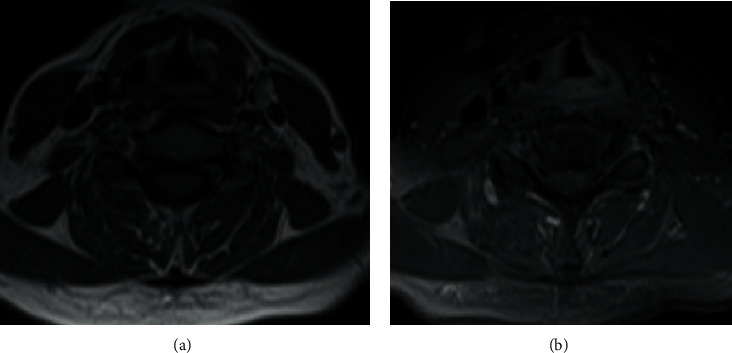
Axial MRI images demonstrating a nonenhancing lesion with fatty marrow changes arising from the left thyroid cartilage. (a) T1 without contrast. (b) T1 fat saturation with contrast.

A 50-year-old female with no significant medical history presented to clinic with nine months of dysphonia and aphonic breaks. Her symptoms progressed gradually and were not associated with any alleviating or aggravating factors. She denied dysphagia, fevers, chills, or weight loss. On physical exam, her voice was found to be mildly rough and breathy, with a mildly reduced pitch range. Laryngovideostroboscopy revealed a left false vocal fold fullness, faster right mucosal wave, and a mild posterior glottal gap (Figure 1(a)). On abduction, there was a subtle fullness of the left midmembranous vocal fold (Figure 1(b)). Neck palpation was normal. A CT neck demonstrated a well-circumscribed, 5 × 8 × 9 millimeter mass arising from the left thyroid cartilage lamina with a thin calcified rim and a heterogeneous decreased attenuation center, suspicious for a chondroma versus low-grade chondrosarcoma (Figure 2). An MRI neck showed a 5 × 7 millimeter left thyroid cartilage lesion with fatty marrow (Figure 3).

**Figure 4:**
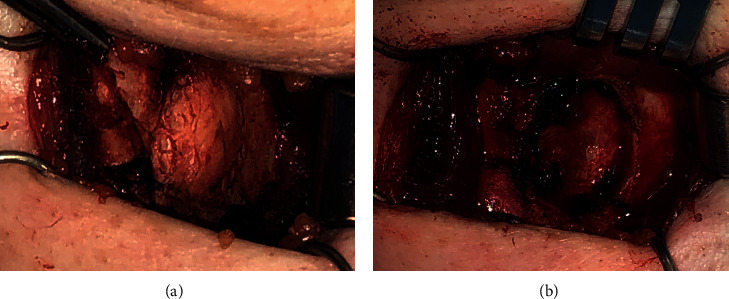
(a) Ovoid, vertically oriented mass within the lamina of the left thyroid cartilage. (b) Defect in thyroid cartilage after removal of mass. The inner perichondrium was left intact.

**Figure 5:**
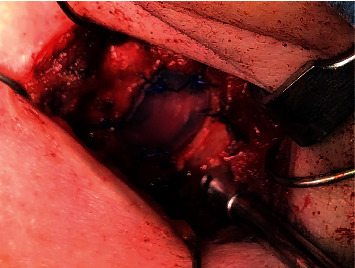
A silastic block trimmed to fit into the thyroid cartilage defect and sutured in place using a series of interrupted 4-0 prolene sutures.

For definitive diagnosis of the mass and management of her dysphonia, the patient was taken to the operating room for surgical excision. The tumor was excised from the thyroid cartilage with clear margins, and the inner perichondrium was left intact (Figure 4). The ovoid defect in the thyroid cartilage was reconstructed with a silastic block that was the same thickness and shape of the excised thyroid cartilage and was sutured to the remaining thyroid ala (Figure 5). Histopathologic analysis of the tumor demonstrated hyaline cartilage overlying lamellar bone with fatty bone marrow, consistent with osteochondroma [1].

## 3. Discussion

Osteochondromas are relatively common benign bone tumors; however, they typically are found on the surface of long bones. In this report, we present a rare case of an osteochondroma arising from the left thyroid cartilage lamina. There have been no reported cases of osteochondromas arising from the thyroid cartilage, and only seven reported cases of osteochondromas of the laryngeal framework [4]. The presenting features of osteochondromas typically depend on the size and location of the lesion. This patient's presentation of insidious dysphonia is consistent with a slow-growing mass on the thyroid cartilage.

The differential diagnosis of a thyroid cartilage mass is broad and includes pathologies such as chondroma/enchondroma, periosteal osteosarcoma, and chondrosarcoma, among others. The diagnosis of chondroid tumors is typically made based on the characteristic imaging findings. Radiographic features of osteochondroma include endosteal scalloping, thick periosteal reaction, and cortical hook seen on CT or MRI [2]. Histologically, these tumors are composed of a cartilaginous cap overlying a pedunculated or sessile base of bone [1]. Enchondroma is a benign cartilaginous tumor that classically presents with stippled calcification, endosteal scalloping, and areas of ossification or expanded cortex on imaging [2]. Unlike osteochondroma, chondromas lack bony features on histology and closely resemble hyaline cartilage [1].

It is critical to distinguish benign tumors from aggressive malignancies such as chondrosarcoma or periosteal osteosarcoma. Radiographic features concerning for malignancy include heterogeneous density and irregular margins suspicious for periosteal osteosarcoma, and ring-and-arc calcifications and cortical erosion suspicious for chondrosarcoma [5]. In this case, the preoperative imaging could not differentiate between a benign or malignant lesion, so histopathologic analysis was required for definitive diagnosis. The surgical specimen findings of hyaline cartilage overlying lamellar bone with fatty bone marrow were diagnostic for osteochondroma [1].

Osteochondromas do not need to be treated unless they are causing symptoms such as pain, deformity, pathologic fractures, or mobility issues. In symptomatic patients, the preferred treatment is surgical excision. This patient suffered from chronic dysphonia in the presence of a thyroid cartilage mass, so the decision was made to take her to the operating room for tumor excision. Due to the benign nature of her lesion, complete excision of the mass was adequate, and no additional treatment was indicated.

## 4. Conclusion

In this report, we describe a rare case of thyroid cartilage osteochondromacausing mild dysphonia. Osteochondromas are relatively common benign bone tumors that classically affect long bones; however, this is the first known report of this tumor affecting the thyroid cartilage. It is prudent to consider osteochondromas on the differential diagnosis of a bony laryngeal mass, and it is important to distinguish them from malignancies in order to guide appropriate therapy.

## Data Availability

The data used to support the findings of this study are included within the article.
